# Dressing Wear Time after Breast Reconstruction: A Randomized Clinical Trial

**DOI:** 10.1371/journal.pone.0166356

**Published:** 2016-12-02

**Authors:** Daniela Francescato Veiga, Carlos Américo Veiga Damasceno, Joel Veiga-Filho, Luiz Francisley Paiva, Fernando Elias Martins Fonseca, Isaías Vieira Cabral, Natália Lana Larcher Pinto, Yara Juliano, Lydia Masako Ferreira

**Affiliations:** 1 Division of Plastic Surgery, Department of Surgery, Universidade Federal de São Paulo, São Paulo, São Paulo, Brazil; 2 Division of Plastic Surgery, Department of Surgery, Universidade do Vale do Sapucaí, Pouso Alegre, Minas Gerais, Brazil; 3 Department of Microbiology, Universidade do Vale do Sapucaí, Pouso Alegre, Minas Gerais, Brazil; 4 Department of Bioestatistics, Universidade do Vale do Sapucaí, Pouso Alegre, Minas Gerais, Brazil; 5 Department of Bioestatistics, Universidade Federal de São Paulo, São Paulo, São Paulo, Brazil; Rabin Medical Center, ISRAEL

## Abstract

**Background:**

The evidence to support dressing standards for breast surgery wounds is empiric and scarce.

**Objective:**

This two-arm randomized clinical trial was designed to assess the effect of dressing wear time on surgical site infection (SSI) rates, skin colonization and patient perceptions.

**Methods:**

A total of 200 breast cancer patients undergoing breast reconstruction were prospectively enrolled. Patients were randomly allocated to group I (dressing removed on the first postoperative day, n = 100) or group II (dressing removed on the sixth postoperative day, n = 100). SSIs were defined and classified according to criteria from the Centers for Disease Control and Prevention. Samples collected before placing the dressing and after 1 day (group I) and 6 days (both groups) were cultured for skin colonization assessments. Patients preferences and perceptions with regard to safety, comfort and convenience were recorded and analyzed.

**Results:**

A total of 186 patients completed the follow-up. The global SSI rate was 4.5%. Six patients in group I and three in group II had SSI (p = 0.497). Before dressing, the groups were similar with regard to skin colonization. At the sixth day, there was a higher colonization by coagulase-negative staphylococci in group I (p<0.0001). Patients preferred to keep dressing for six days (p<0.0001), and considered this a safer choice (p<0.05).

**Conclusions:**

Despite group I had a higher skin colonization by coagulase-negative staphylococci on the sixth postoperative day, there was no difference in SSI rates. Patients preferred keeping dressing for six days and considered it a safer choice.

**Trial Registration:**

ClinicalTrials.gov NCT01148823

## Background

Owing to its numerous and varied psychosocial benefits, breast reconstruction is currently considered to be an integral part of breast cancer treatment [1]. However, surgical site infection (SSI) is a major cause of postoperative morbidity after breast reconstruction. Despite being considered a clean procedure, breast cancer surgery has been associated with higher SSI rates than would be expected for other clean surgical procedures [2]. These rates vary widely in literature, with reported rates of up to 53% [3,4–9].

SSI often leads to longer hospital stay, hospital readmission, reoperation, reconstructive failure, implant loss, delay of postoperative adjuvant therapies and psychosocial distress [3,10–12]. Furthermore, SSI can increase the costs associated with the surgery and decrease patient satisfaction after surgery [13,14]. Thus, interventions to reduce the incidence of SSI are essential to reduce not only morbidity, but also costs to the patient and the society [11,13–19].

Risk factors for SSIs can be classified into preoperative (patient-related), perioperative (procedure-related) and postoperative categories [15,20]. Wound management plays a major role in mitigating SSI risk factors in the postoperative period [15,20–22]. However, literature on incisional wound management is scarce, with many issues that need to be clarified [22–24].

The global market for wound dressings is growing, as new materials and application techniques are developed [25–27]. However, there is no solid evidence to guide product choice [28,29] and traditional low-technology gauze-based dressings are commonly used [30–32]. This kind of dressing is widely used in the public health system in Brazil due to its low cost.

There is also no consensus in literature for how long surgical dressings should be worn. Some authors recommend the early exposure of the wound [22,23,33], whereas others recommend keeping dressings in place for longer periods, until the sutures or drains are removed [16,24,31].

The Centers for Disease Control and Prevention (CDC) guidelines for the management of surgical wounds that are primarily closed instruct that patients should keep their wounds dry and covered with a sterile dressing for 24–48 hours [20]. There are no recommendations regarding the type of dressing or the length of time that the dressing should be worn after breast surgery. Thus, decisions are made empirically, based on the surgeon's personal experience.

The current standard in the hospital where the present trial was performed is to remove the dressing 24 hours after breast reconstruction. In a previous study, we found less colonization by coagulase-negative staphylococci after reduction mammaplasty when dressings were kept in place longer (for 6 days), without a change in the SSI rate [34]. The results of that trial led us to hypothesize that keeping the dressing in place longer than the 24–48 hours recommended by CDC would be beneficial for breast reconstruction patients. However, those findings may not apply to breast reconstruction patients, due to several reasons: breast reconstruction is applied to cancer patients, not to healthy breast hypertrophy women, implants are frequently used, and the operation time is many times longer than that of reduction mammaplasty, which may affect the optimal dressing time.

To the best of our knowledge, this is the first randomized trial assessing the time that surgical dressing should be worn after breast reconstruction. Our primary objective was to assess the effect of longer surgical dressing wear time on SSI rates. Secondary aims were to assess skin bacterial colonization and to determine patient preferences and perceptions regarding the safety, comfort and convenience of surgical dressing wear times.

## Methods

### Study design, ethics and setting

This is a two-arm parallel group randomized clinical trial. The study protocol has been published [35].

This trial was conducted according to the principles expressed in the Declaration of Helsinki. The Institutional Ethics Committee approved the study protocol (Ethics Committee of the Universidade do Vale do Sapucaí, 786/07 and 1623/11). Participants were included after providing written informed consent.

In October 2007, the Ethics Committee approved an initial protocol for a study of patients undergoing various kinds of clean plastic surgery procedures of the thorax, including breast reconstruction. However, owing to the great heterogeneity of procedures, we were unable to attribute any differences in SSI rates, skin colonization or patient perceptions to the length of time that the dressing was kept in place. Therefore, another randomization in blocks of 40 patients was generated in 2009, with patients stratified by surgical procedure. Only breast reconstruction patients, recruited after the stratification, were included in the present trial.

Recruitment for the present trial began in June 2009. After 18 months of follow-up, with the stratified sample, we realized that we could not compare different surgical procedures. Therefore, the study was split, and the sample size was recalculated for breast reconstruction surgery specifically. The Ethics Committee approved the breast reconstruction trial in June 2011. Because all procedures remained rigorously the same, the 40 breast reconstruction patients who had been recruited in 2009 remained in the analysis.

Patients were recruited and followed-up at the Breast Unit of the Plastic Surgery Division of a university-affiliated hospital, the Hospital das Clínicas Samuel Libânio–Universidade do Vale do Sapucaí.

### Sample size

In our hospital, the average rate of SSI after breast reconstruction was 4% over the last 10 years. A 10% difference in SSI rate was considered clinically relevant [17,34,36]. A one-tailed hypothesis test for two population proportions was performed (α = 0.05, β = 0.2 or 80% power) to identify differences in SSI rate between groups I and II. The estimated sample size was 100 patients per arm.

### Eligibility criteria and group assignment

Breast cancer patients aged 18 years and older who were undergoing immediate or delayed breast reconstruction by any technique were considered eligible for participation. Patients who smoked heavily, had a body mass index (BMI) above 35kg/m^2^, were diabetic, who had any contraindication for breast reconstruction procedures were excluded, as were patients whose dressing had gotten wet in the first 24 hours after the operation.

As described previously [35], 200 patients were randomly assigned to group I (n = 100), with dressings removed on the first postoperative day, or to group II (n = 100), with dressings removed on the sixth postoperative day.

The allocation was determined by a computer-generated sequence (Bioestat 5.0, Instituto de Desenvolvimento Sustentável Mamirauá, Belém, PA, Brazil). A sealed, opaque, sequentially numbered envelope was opened on the first postoperative day to reveal if the patient was assigned to group I or group II [35].

DFV generated the random allocation sequence. DFV and JVF recruited and selected participants, FEMF, IVC and NLLP assigned participants to interventions.

### Procedures and interventions

Before admission to the operating unit, all patients were instructed to shower with a liquid detergent-based 4% chlorhexidine [37]. An alcoholic 0.5% chlorhexidine solution was used for antisepsis of the surgical site [38]. When an immediate breast reconstruction was performed, the same antiseptic solution was reapplied to the surgical site after the oncologic operation, immediately before breast reconstruction.

All participants underwent immediate or delayed breast reconstruction, using flaps and/or implants. All operations were performed under general anesthesia by the same surgical team. None of the patients underwent bilateral reconstruction. A drain was used in most patients and was removed when the discharge was less than 50mL/day.

All patients received 1g of cephalotin administered intravenously at the induction of anesthesia and every 6 hours postoperatively, for the first 24 hours. After 24 hours, patients received 500mg of cephalexin, which was administered orally four times per day, prescribed for six days.

As described in details in our previously published work [34,35,39], at the end of the operation, the surgical site was cleaned with sterile physiological saline and swabbed for culture. Surgical wounds were covered with four layers of dry sterile cotton gauze. The wounds were completely covered, and the gauze layers were fixed in place by a micropore tape. The surgical team was blinded with regard to the patient allocation into groups.

After 24 hours, the patient group was revealed by opening the sealed envelope with the patient’s study number. As previously described [34,35,39], patients allocated to group I were instructed to keep their wounds uncovered and to follow their usual personal hygiene routine. Patients in group II were instructed not to wet the dressing.

Depending on the breast reconstruction technique used and on patient's clinical conditions, patients were discharged from the hospital within 1 to 5 days. To monitor SSI, all patients were instructed to return for weekly follow-ups for 30 days or at any time after 30 days if they noticed any signs or symptom of infection, such as localized pain, swelling, redness, wound disruption, or wound drainage. Patients who received an implant were instructed to return again after 1 year for follow-up. When a SSI was diagnosed and there were fluids or purulence, a sample was collected to bacterial culture and antibiogram.

### Outcomes measures

The primary outcome variable was SSI. Secondary outcome variables were skin colonization, as well as patient preferences and perceptions with regard to safety, comfort and convenience [34,35,39].

### Assessment of SSI

The CDC definitions and classifications of SSI were used. An infection was classified as a superficial incisional SSI when it involved only skin or subcutaneous tissue, a deep incisional SSI when it involved deep soft tissues (fascial and muscle layers), or an organ/space SSI when it involved any other part of the anatomy (organs or spaces) [40].

Infections that appeared to be related to the operation were considered SSIs if they occurred within 30 days (for surgeries without implants) or within 1 year (for surgeries with implants) after the operation [40]. A surgeon’s diagnosis of infection was considered an acceptable criterion for an SSI [40].

### Skin colonization

The microbiologic methods used were described in detail in our previous publications [34,35,39]. Samples for skin cultures were obtained immediately before placing the dressing and immediately after dressing removal. In group I, an additional sample was collected on the sixth postoperative day. A standard 5 x 10 cm area over the breast surgical wound was swabbed with two sterile cotton swabs that had been pre-moistened with sterile saline. Each swab was placed in a sterile container with 1.0 mL of saline and immediately taken to the laboratory.

Standard microbiologic methods were used [41]. As we previously described [34,35,39], 0.2mL aliquots of the first sample were plated on hypertonic manitol (HM) agar, on Sabouraud agar with chloramphenicol (0.05mg/mL), and on eosin-methylene blue (EMB) agar, selective for staphylococci, fungi and enterobacteria, respectively. An aliquot of the first sample was also plated on blood agar, to identify hemolytic colonies. Plates were incubated in an aerobic atmosphere, at 37°C, for 48 hours (blood agar and EMB agar), 4 days (HM agar) or 7 days (Sabouraud agar).

Aliquots of 0.5 mL of the second sample were inoculated into thioglycolate broth and glucose broth. The solutions were incubated aerobically at 37°C and checked after 72 hours. They were incubated until they turned positive, at a maximum of seven days. Two blinded laboratory technicians processed all samples. After the incubation period, plates and tubes were examined by a single microbiologist, who was also blinded to patient grouping.

As previously described [34,35,39], culture results were reported as colony forming units (CFUs) per plate. Whenever the number of CFUs in a plate exceeded 300, it was recorded as over 300. Staphylococci were identified as coagulase-negative *Staphylococcus* sp. or *S*. *aureus* by means of the Gram stain, the presence of hemolysis and the coagulase testing. Cultures in thioglycolate and glucose broths were reported as positive or negative.

### Patient perceptions

The preferences and perceptions of patients were assessed at on their 2-week postoperative follow-up visit. Preferences and perceptions were assessed by using a previously published self-assessment instrument, which scores patients perceptions of safety, comfort and convenience related to their dressing wear time on a 5-point rating scale. This self-administered instrument also includes a question about the patient's preference for wear time (1 day or 6 days) [34,35,39].

### Exit points

As described in the study protocol [35], participation was considered complete after the postoperative assessment on day 30 (if no implants were used) or after the 12^th^ postoperative month (if an implant was used) [20]. Other exit points included getting the dressing wet before the sixth postoperative day, in group II, requiring withdrawal from the study, and not returning for follow-up assessments.

### Blinding

The surgical team, including who applied the dressing, the laboratory technicians who processed the samples and the microbiologist were blinded to patient grouping. It was not possible to blind patients and the surgeon who assessed SSI.

### Statistical analysis

Due to the nature of the variables studied, non-parametric statistics were used [42]. The rejection level for the null hypothesis was fixed at 5% (p≤ 0.05).

The Mann-Whitney U test was used to compare age and BMI of patients groups I and II, as well as duration of operations. Chi-square tests were applied to compare groups I and II with regard to the occurrence of SSI, the time of breast reconstruction (immediate/delayed), the technique of breast reconstruction, the microorganism growth in thioglycolate and glucose broths, and the patient preference for the dressing wear time (1 day / 6 days), as well as to verify associations between the occurrence of SSI and the technique of breast reconstruction. Logistic regressions were applied to detect significant associations between the occurrence of SSI and variables such as age, BMI and duration of operation.

A Friedman two-way analysis of variance was used to assess differences in the number of CFUs among the three time points (pre-dressing, first and sixth postoperative days), in group I. Whenever the difference was significant, multiple comparisons tests were performed to determine which time points were significantly different [42]. A Wilcoxon signed-rank test was applied to compare the number of CFUs before dressing and on the sixth postoperative day, in group II. A Mann-Whitney U test was used to compare skin colonization in groups I and II before dressing and on the sixth postoperative day. The tests were always applied independently for each medium used.

The Fisher Exact Test was used to compare groups I and II with regard to the growth of *S*. *aureus*. The Kolmogorov-Smirnov test was used to compare the two groups regarding patients’ perceptions of safety, comfort and convenience.

Statistical analysis was performed using SPSS (*Statistical Package for Social Sciences*, Inc., Chicago, Ill.) v.18 and Bioestat 5.0 (Instituto de Desenvolvimento Sustentável Mamirauá, Belém, PA, Brazil).

## Results

**Fig 1:**
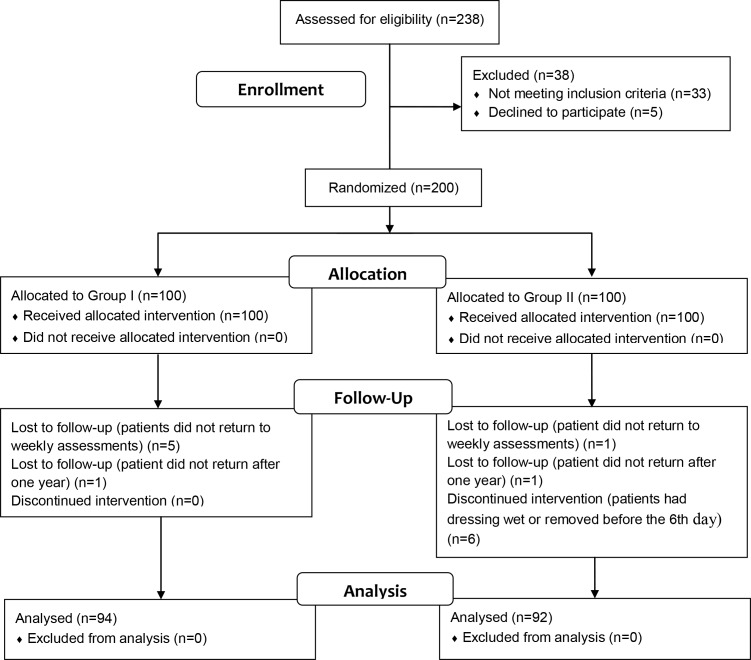
CONSORT Flow Diagram [43]

Patients were recruited between June 2009 and September 2013, and follow-up finished on October 2014. A total of 186 (93%) patients completed the study. Fig 1 presents the participants flow diagram [43].

At baseline, groups were matched for age, BMI and duration of operation (Table 1). None of these variables was associated with a higher incidence of SSI (p = 0.214, p = 0.742 and p = 0.134, respectively). Groups were also matched for time of breast reconstruction and technique used (Table 2).

**Table 1:** Demographic and surgical variables of patients in both groups and comparison by Mann-Whitney U test

**Group I** (n = 100)			
	**Age** (years)	**BMI**^**a**^ (Kg/m^2^)	**Duration of operation** (minutes)
Range	23–70	19.1–30.8	50–300
Median ± IQR^b^	48 ± 12	25.8 ± 4.7	100 ± 40
Mean ± SD^c^	47.8 ± 9.5	25.8 ± 2.9	122.1 ± 62.8
**Group II** (n = 100)			
Range	29–70	20.3–30.0	50–300
Median ± IQR^b^	50 ± 13	24.9 ± 4.6	90 ± 50
Mean ± SD^c^	49.3 ± 9.3	25.2 ± 2.9	110.2 ± 57.3
**Group I *vs*. Group II** (Mann-Whitney U test)			
	p = 0.200	p = 0.075	p = 0.094

^a^BMI: body mass index

^b^IQR: interquartile range

^c^SD: standard deviation

**Table 2:** Time and technique of breast reconstruction and comparison between group I and group II by Chi-square test

Time	n		
	Group I (n = 100)	Group II (n = 100)	Group I *vs*. Group II (Chi-square test)
Immediate	58	48	p = 0.157
Delayed	42	52	
**Technique**			
Tissue expander / Prosthesis	57	65	p = 0.327
Latissimus dorsi flap	4	6	
TRAM flap	14	7	
Local or regional flaps	25	22	

Nine (4.5%) patients developed SSI, six from group I and three from group II. The difference between groups was not statistically significant (p = 0.497). Six of these patients had undergone delayed breast reconstruction, and three had undergone immediate reconstruction. Six patients had tissue expander reconstruction, two patients underwent latissimus dorsi myocutaneous flap reconstruction and one patient had a TRAM flap reconstruction. Groups I and II each had no association between the breast reconstruction technique used and SSI occurrence. However, in an overall analysis including both groups, there was a significant association with latissimus dorsi flap reconstruction (p = 0.0410, Table 3).

**Table 3:** Association between technique of breast reconstruction and SSI occurrence, in groups I and II and overall, by Chi-square test

	SSI (n)					
	Group I (n = 94)		Group II (n = 92)		Overall (n = 186)	
Technique	Yes	No	Yes	No	Yes	No
Tissue expander / Prosthesis	4	49	2	58	6	107
Latissimus dorsi flap	1	3	1	4	2	7
TRAM flap	1	12	-	6	1	18
Local or regional flaps	-	24	-	21	-	45
**SSI—Yes *vs*. No** (Chi-square test)	p = 0.249		p = 0.148		p = 0.041	

Five patients had deep incisional SSIs, four from group I and one from group II. The other four patients had superficial incisional SSIs, involving only skin or subcutaneous tissue of the incision [40]. All five patients with deep incisional SSI had undergone implant reconstruction and required readmission to the hospital to remove the breast implant. In the other cases, SSI was successfully treated with oral antibiotics, and none of these patients was readmitted to the hospital.

Time to onset of SSI ranged from 2 to 13 weeks (median ± interquartile range: 3±1.5 weeks) in group I, and from 2 to 9 weeks (median ± interquartile range: 3±3.5 weeks) in group II.

Group I had significantly higher skin colonization on the sixth postoperative day (Table 4). Descriptive statistics of CFUs counts and statistical comparisons intragroup and intergroups are presented in Table 5.

**Table 4:** Microorganisms growth in thioglycolate broth and glucose broth and comparison between group I and group II by Chi-square test

	n (%)	
Thioglycolate broth (+)^a^	Pre dressing (n = 100)	Postoperative day 6 (n = 94 / n = 92)
Group I	26 (26.0)	39 (41.5)
Group II	27 (27.0)	21 (22.8)
**Group I *vs*. Group II** (Chi-square test)	p = 0.873	p = 0.006
**Glucose broth (+)**^**a**^		
Group I	28 (28.0)	44 (46.8)
Group II	30 (30.0)	24 (26.1)
**Group I *vs*. Group II** (Chi-square test)	p = 0.755	p = 0.003

^a^(+): positive culture

**Table 5:** Skin colonization results

	**Number of Colony Forming Units**			
**Group I**	**HM**^a^ **agar**	**Blood agar**	**EMB**^**b**^ **agar**	**Sabouraud agar**
**Pre dressing** (n = 100)				
Range	0–300	0–300	0–5	0–26
Median ± IQR^c^	0 ± 0	0 ± 0	0 ± 0	0 ± 0
Mean ± SD^d^	4.5 ± 30.1	5.4 ± 30.5	0.1 ± 0.5	0.3 ± 2.6
**Postoperative day 1** (n = 100)				
Range	0–300	0–300	0–5	0–17
Median ± IQR^c^	0 ± 0	0 ± 0	0 ± 0	0 ± 0
Mean ± SD^d^	4.2 ± 30.4	4.5 ± 31.2	0.1 ± 0.5	0.2 ± 1.8
**Postoperative day 6** (n = 94)				
Range	0–300	0–300	0–300	0–300
Median ± IQR^c^	4 ± 30.8	2 ± 34.8	0 ± 0	0 ± 0
Mean ± SD^d^	40.6 ± 85.6	43.7 ± 87.7	7.5 ± 43.8	3.3 ± 30.9
**Pre *vs*. day 1 *vs*. day 6** (Friedman test)	p = 0.000 Pre and day 1 < day 6	p < 0.0001 Pre and day 1 < day 6	p = 0.713	p = 0.976
**Group II**				
**Pre dressing** (n = 100)				
Range	0–86	0–300	0–51	0–0
Median ± IQR^c^	0 ± 0	0 ± 0	0 ± 0	0 ± 0
Mean ± SD^d^	3.8 ± 13.9	8.2 ± 35.1	0.5 ± 5.1	0 ± 0
**Postoperative day 6** (n = 92)				
Range	0–300	0–300	0–0	0–0
Median ± IQR^c^	0 ± 0	0 ± 0	0 ± 0	0 ± 0
Mean ± SD^d^	8.9 ± 44.5	11.4 ± 48.6	0 ± 0	0 ± 0
**Pre *vs*. day 6** (Wilcoxon test)	p = 0.879	p = 0.811	p = 0.317	-
**Group I *vs*. Group II** (Mann-Whitney U test)				
**Pre dressing**	p = 0.883	p = 0.875	p = 0.999	p = 0.807
**Postoperative day 6**	p < 0.000 Group I > Group II	p < 0.0001 Group I > Group II	p = 0.380	p = 0.616

^a^HM: hypertonic manitol

^b^EMB: eosin-methylene blue

^c^IQR: interquartile range

^d^SD: standard deviation

*S*. *aureus* was recovered from samples of nine patients (4.5%), seven from group I and two from group II (p = 0.169). Among the seven patients in group I with *S*. *aureus*, four developed SSI. Neither of the other patients with positive *S*. *aureus* cultures, in both groups, developed SSI. Of the nine cases of SSI in the current study, *S*. *aureus* was involved in four (44%), whereas *S*. *epidermidis* was involved in one (11%).

Overall, 163 (81.5%) patients completed the patient perceptions assessment, 82 from group I and 81 from group II. Table 6 presents patient perceptions of safety, comfort and convenience related to their dressing wear time. Patients preferred to keep dressing on for 6 days (p<0.0001, Table 7).

**Table 6:** Perceptions of patients in group I (n = 82) and group II (n = 81) related to their dressing wear time

	Safety n (%)		Comfort n (%)		Convenience n (%)	
	Group I	Group II	Group I	Group II	Group I	Group II
Excellent	42 (51)	64 (79)	43 (52)	46 (57)	59 (72)	46 (57)
Very good	23 (28)	13 (16)	24 (29)	20 (25)	20 (24)	20 (25)
Good	17 (21)	4 (5)	15 (18)	15 (18)	3 (4)	15 (18)
**Group I *vs*. Group II** (Kolmogorov-Smirnov test)	p < 0.05		p > 0.05		p > 0.05	

**Table 7:** Patients’ preferences for dressing wear time and comparison between groups I and II by Chi-square test

	Preference	
	n (%)	
	1 Day	6 Days
**Group I** (n = 82)	68 (82.9)	14 (17.1)
**Group II** (n = 81)	7 (8.6)	74 (91.4)
**Group I *vs*. Group II** (Chi-square test)	p < 0.0001	

## Discussion

SSI prevention has gained worldwide attention because of the associated medical costs and morbidity [11,14,18]. Most hospital readmissions after surgery are due to SSI [1]. With the recent focus on readmission rates as an indicator of quality of care, to minimize the risk of patient readmission after surgery is imperative [43]. Although postoperative wound management is essential for preventing SSI, few studies have addressed this topic [22,24,34].

Theoretically, the greater the degree of surgical wound contamination is, the higher the risk of SSI is [18,20]. In the current study, keeping the dressing in place for 6 days led to lower skin colonization by coagulase-negative staphylococci, but there was no statistical difference between the groups with regard to the growth of *S*. *aureus*. *S*. *epidermidis* are the most common coagulase-negative staphylococci, and have been described as the bacteria most frequently involved in SSI after breast operations [8]. However, many other authors reported *S*. *aureus* as the most common pathogen causing breast surgery SSI [6,10,12,17,23,30]. Our results support these findings. *S*. *epidermidis* was the microorganism most frequently recovered from our samples, but was only involved in one case of SSI. *S*. *aureus* was involved in four of the nine (44%) observed cases of SSI, while *S*. *epidermidis* was involved in one case (11%). Although there was an increase in coagulase-negative staphylococci colonization with the shorter dressing time, the increased colonization rate was not correlated with a significant increase in SSI rates.

The lack of correlation between skin colonization and SSI rates observed in the present trial may be due to the healing process of properly closed clean incisional wounds. Immediately after the incision, an inflammatory process begins with the release of inflammatory mediators that causes vasodilatation. The increased blood flow to the region results in an influx of neutrophils and macrophages, which play a role in digesting bacteria. Simultaneously, platelets adhere to exposed collagen and release the contents of their granules. Platelets and the coagulation cascade are activated by tissue factor, resulting in a fibrin-platelet matrix, which may prevent the invasion of microorganisms into the surgical site [44, 45]. When the wound is properly closed, the process of reepithelialization occurs quickly, also preventing bacteria from entering the surgical wound, regardless of whether the dressing is removed after 1 or 6 days. However, the methods used in this trial did not allow us to characterize the mechanism, and further investigation is needed.

In contrast to previous studies, we found no associations between age, BMI, or duration of operation and SSI rates [1,2,7,15,21,36]. Our SSI rate, of 4.5%, was also relatively low. We speculate that this could be due to the prolonged use of antibiotics. When performing breast surgery, if no implants are used, most American surgeons give intravenous cephalosporin preoperatively, stopping antibiotics right after surgery [46]. Brazilian breast surgeons do the same. However, when a reconstructive procedure is performed, particularly when an implant is placed, plastic surgeons are concerned that SSI will lead to reconstruction failure [2,46]. Indeed, women undergoing breast reconstruction with implants are up to ten times more likely to develop an SSI than patients undergoing aesthetic implant operations [4]. Thus, in these cases, plastic surgeons broadly support the prolonged use of antibiotics [2,17].

Two recent papers assessed the use of antibiotics following breast reconstruction, and both concluded that further studies are needed to clarify this issue [47,48]. In our hospital, it is standardized the use of cephalosporin for seven days following breast reconstruction, administered intravenously at the induction of anesthesia and for 24 hours and subsequently administered orally for additional six days, if no infectious complications occur. Thus, this antibiotic regimen was implemented in our trial.

Korol et al., in a systematic review of risk factors associated with SSI among different surgical procedures, found a time until onset of SSI ranging from 6.2 to 41.4 days post-surgery, with a median overall time of 17 days. They observed that the time until onset tended to be highest in orthopedic surgery and transplant operations, probably due to the risk of delayed infection associated with implants [19]. Our results support these findings, as our overall time until SSI onset was high (median: 3 weeks, range: 2–13 weeks) and 89% of patients (8/9) who developed an SSI (89%) had an implant. We also found a significant association between latissimus dorsi flap reconstruction, which includes an implant, and the occurrence of SSI. However, this association could be due to the small number of latissimus dorsi flap reconstructions performed in the present study. Of the nine latissimus dorsi flap reconstructions performed, two (22.2%) resulted in an SSI, which is a high proportion.

In the current evidence-based medicine era, patient-reported outcomes assessment is a key component of evaluating the success of any procedure. Patient preferences and tolerance must be considered in these assessments [29, 49]. As we hypothesized in the published study protocol [35], the disfiguring characteristic of breast cancer surgical treatment could lead patients to prefer to keep the dressing in place for a longer period, delaying the moment of seeing their reconstructed breasts. Indeed, we found that patients significantly preferred having the dressings in place for 6 days and perceived it as a safer choice.

This trial had several limitations, which should be considered when interpreting its results. This was a single-center study, and all the operations were performed by the same surgical team. Thus, the specific practice style of the center, such as antibiotics use standards and surgical team abilities may have influenced the results, limiting their generalizability. Another limitation was the non-inclusion of patients who had a wet dressing in the first 24 hours, requiring the change of the dressing, because these patients might have been more be prone to SSI occurrence.

## Conclusions

To date, there are no recommendations for the type of dressing or for the ideal dressing wear time after breast surgery. The current trial did not demonstrate any significant effect of dressing wear time on SSI rates after breast reconstruction, although keeping the dressing in place for 6 days led to lower skin colonization by coagulase-negative staphylococci. However, patients preferred to keep the dressing on for 6 days and considered this a safer choice. Therefore keeping the dressing in place for a longer time, beyond the 24–48 hours currently recommended, may be beneficial for breast reconstruction patients. Further multi-center studies, with larger sample sizes, are necessary to reach a conclusive answer regarding dressing wear time after breast reconstruction.

## Supporting Information

S1 File Original Study Protocol in Portuguese (DOC)

S2 File Original Study Protocol in English (DOC)

S1 Table Group I Dataset (XLS)

S2 Table Group II Dataset (XLS)

S3 Table CONSORT Checklist (DOC)
