# Supplementary material for: Dressing Wear Time after Breast Reconstruction: A Randomized Clinical Trial
Source: PLoS One. 2016 Dec 2;11(12):e0166356. doi: 10.1371/journal.pone.0166356 (PMC5135046; doi:10.1371/journal.pone.0166356)

**TEMPO DE MANUTENÇÃO DO CURATIVO APÓS RECONSTRUÇÃO MAMÁRIA: INFLUÊNCIA DA COLONIZAÇÃO CUTÂNEA E OCORRÊNCIA DE INFECÇÃO DO SÍTIO CIRÚRGICO**

1. **RESUMO**

**Local:** Projeto a ser desenvolvido no Programa de Pós-Graduação em Cirurgia Plástica da Universidade Federal de São Paulo – UNIFESP e na Universidade do Vale do Sapucaí – UNIVÁS

**Contexto:** Infecção da ferida operatória permanece um problema importante com muitos pontos a serem elucidados, e persiste como a maior causa de morbidade no paciente cirúrgico. Embora o cuidado com a ferida operatória faça parte do cotidiano do cirurgião, a conduta adotada no seu manejo é baseada em escassa evidência científica, e o tempo de manutenção do curativo é baseado na tradição e não em evidências.

**Objetivos:** Avaliar a influência do tempo de manutenção do curativo após reconstrução mamária sobre a taxa de infecção do sítio cirúrgico e a colonização cutânea, bem como a percepção das pacientes sobre o tempo de cobertura da ferida operatória.

**Métodos:** Trata-se de um ensaio clínico, prospectivo, randomizado. Serão selecionadas, consecutivamente, 200 mulheres com câncer de mama ou previamente submetidas a tratamento cirúrgico do câncer mamário, candidatas a reconstrução mamária imediata ou tardia. As pacientes serão randomicamente alocadas para o grupo PO1 (n=100), em que a ferida operatória ficará coberta por um dia ou para o grupo PO6 (n=100), em que a ferida ficará coberta por seis dias. Serão coletadas amostras para culturas no momento pré-curativo e imediatamente após a remoção do curativo, para avaliação da colonização cutânea. Para avaliação da ocorrência de infecção do sítio cirúrgico serão utilizados os critérios e definições do *Centers for Disease Control and Prevention*. As pacientes serão avaliadas semanalmente, por 30 dias, e as pacientes submetidas à inclusão de implantes serão reavaliadas após um ano. A percepção das pacientes será avaliada por meio de instrumento estudo-específico. Os dados obtidos serão submetidos à análise estatística.

**Palavras-chave:** câncer mamário, reconstrução da mama, cuidados pós-operatórios, curativos, infecção pós-operatória, crescimento bacteriano

1. **INTRODUÇÃO**

Infecção da ferida operatória permanece um problema importante com muitos pontos a serem elucidados, e persiste como a maior causa de morbidade no paciente cirúrgico.^1,2^ A ocorrência estimada de infecção da ferida operatória no Brasil é em torno de 11% das cirurgias realizadas.^3^ Nos Estados Unidos da América, a infecção da ferida operatória é responsável por um quarto das infecções hospitalares, resultando em prolongamento do período de internação hospitalar e aumento de custos.^4^

O risco de infecção para cirurgias limpas é estimado em 1 a 2%.^5^ As taxas de infecção após cirurgias mamárias parecem ser muito maiores do que o esperado para cirurgias limpas.^6-8^ A incidência de infecção após mastectomia varia de 2,8% a 25% na literatura,^6,9-11^ e as taxas de infecção após reconstrução mamária variam de 6,3% a 28%.^6,12-16^ Entender os fatores de risco para infecção após cirurgias mamárias é essencial para o desenvolvimento de estratégias de prevenção.^12^

Os fatores de risco para infecção da ferida operatória geralmente são separados em três categorias: os relacionados ao paciente (pré-operatórios), os relacionados ao procedimento (intra-operatórios) e os relacionados ao pós-operatório.^17^ Os fatores relacionados ao paciente incluem idade, obesidade, tabagismo, comorbidades, uso de drogas imunosupressoras. Fatores relacionados ao procedimento incluem tipo de procedimento (limpo, potencialmente contaminado, contaminado, infectado), duração da cirurgia, hipóxia, trânsito na sala cirúrgica e parâmetros físicos da sala cirúrgica, entre outros.^17,18^ No período pós-operatório, um dos maiores fatores de risco para infecção é o manejo da ferida operatória.^17-19^

A proteção das feridas tem sido uma ação instintiva do homem; existem evidências de que já era realizada há cerca de 4000 anos.^20^ As razões para o uso de curativos em feridas cirúrgicas incluem proteção mecânica contra traumas, prevenção de contaminação e de perdas de fluídos. Além disso, o curativo proporciona uma melhor aparência da região operada, visando ao bem-estar psicológico do paciente.^21^ O curativo ideal deve proteger a ferida contra trauma e contaminação, absorver secreções, e proporcionar a compressão necessária para minimizar edema e obliterar espaços resultantes do divulsionamento de tecidos.^20,22,23^

A busca por um curativo ideal para cirurgias mamárias tem levado ao desenvolvimento de um grande número de diferentes materiais e técnicas de aplicação.^24,25^ Entretanto, a padronização de curativos para feridas incisionais, incluindo as cirurgias mamárias, é baseada em evidências empíricas e escassas.^19,21-23,26,27^

O tempo ideal de cobertura de feridas incisionais é controverso na literatura. Alguns autores recomendam a remoção precoce do curativo, para facilitar a observação da ferida, liberar o paciente para sua rotina de higiene pessoal e diminuir custos.^22,28,29^ Chrintz *et al.*, em um ensaio clínico aleatório com 1202 pacientes, observaram que a retirada do curativo no primeiro dia pós-operatório não implicou em diferença na taxa de infecção pós-operatória em relação ao grupo em que o curativo foi mantido até a remoção das suturas.^22^

Outros autores preconizam manter o curativo colocado no centro cirúrgico, sob condições ideais de antissepsia, até a remoção das suturas, desde que o mesmo permaneça seco.^28,30-33^ Alvarez demonstrou que o uso de curativo oclusivo por duas semanas, após esternotomia em cirurgias cardíacas, diminuiu a incidência de infecção pós-operatória.^33^ Rosenfeldt *et al.* demonstraram que o curativo oclusivo reduziu a taxa de infecção pós-operatória em cirurgias no membro inferior.^32^

O manejo de feridas cirúrgicas deve envolver o princípio de minimizar danos, e a preferência e tolerância do paciente devem ser consideradas.^26^ Chrintz et al. relataram que retirar o curativo após as primeiras 24 horas tem vantagens, como permitir que os pacientes retomem sua rotina de higiene pessoal mais facilmente.^22^ Por outro lado, outros autores observaram que o paciente se sente mais seguro e confortável com a ferida operatória recoberta pelo curativo.^23,27^

Embora o cuidado com a ferida operatória faça parte do cotidiano do cirurgião, a conduta adotada no seu manejo é baseada em escassa evidência científica, e o tempo de manutenção do curativo é baseado na tradição e não em evidências.^28,29^

O “Guia para Prevenção de Infecção do Sítio Cirúrgico” do *Centers for Disease Control and Prevention* (CDC) recomenda proteger as feridas que tenham sido fechadas primariamente com um curativo estéril por 24 a 48 horas.^18^ Não há nenhuma recomendação para manter uma incisão fechada primariamente coberta por mais de 48 horas, e também não há nenhuma indicação sobre o tempo apropriado para que o paciente tome banho com a incisão descoberta. Estes permanecem tópicos a serem esclarecidos.^18^

Assim, este ensaio clínico aleatório foi desenhado para avaliar a influência do tempo cobertura da ferida operatória sobre a ocorrência de infecção e a colonização cutânea, assim como a percepção das pacientes, que sempre deve ser considerada.

1. **OBJETIVOS**

- 1. **Objetivo primário:**
- Avaliar a influência do tempo de manutenção do curativo após reconstrução mamária sobre a taxa de infecção do sítio cirúrgico.
  1. **Objetivos secundários:**
- Avaliar a influência do tempo de manutenção do curativo sobre a colonização cutânea;
- Avaliar a percepção das pacientes sobre o tempo de manutenção do curativo.

1. **MÉTODOS**
   1. **Tipo de estudo:**

Primário, clínico, prospectivo, randomizado, controlado, intervencional, analítico.

- 1. **Casuística:**
     1. **Cálculo do tamanho:**

Considerando que a proporção de infecção do sítio cirúrgico após reconstrução mamária, na literatura, varia de 2% a 28%,^6,12-16^ e considerando significativa uma diferença de 10 pontos percentuais, o número calculado de pacientes, por grupo, foi de 100, com nível de significância de 5% e poder do teste de 80%.

- - 1. **Seleção:**

Serão selecionadas, consecutivamente, nos ambulatórios de Mastologia e Cirurgia Plástica Mamária do Hospital das Clínicas Samuel Libânio (HCSL), da Universidade do Vale do Sapucaí, Pouso Alegre – MG, 200 pacientes com câncer mamário, candidatas a reconstrução mamária imediata ou que foram previamente submetidas a tratamento cirúrgico do câncer mamário, candidatas a reconstrução mamária tardia. As pacientes que preencherem os critérios de elegibilidade serão esclarecidas sobre o estudo e só serão incluídas caso concordem em participar, assinando o termo de consentimento livre e esclarecido.

Para a seleção das pacientes serão considerados os critérios de elegibilidade relacionados a seguir.

Critérios de inclusão:

- Pacientes do gênero feminino, entre 18 e 70 anos de idade, sem restrição quanto à etnia, escolaridade e classe social;
- Pacientes portadoras ou previamente tratadas de câncer mamário, candidatas à reconstrução mamária.

Critérios de exclusão:

- Comorbidades não controladas que constituam contra-indicação para o procedimento cirúrgico;
- Vigência de tabagismo;
- IMC superior a 35Kg/m^2^;
- Pacientes que tenham o curativo molhado nas primeiras 24h, necessitando sua substituição.
  - 1. **Alocação:**

Para alocação das pacientes nos grupos será gerada seqüência aleatória pelo *software* Bioestat 5.0 (Instituto Mamirauá, Brasil), e o sigilo de alocação será garantido por envelopes opacos selados abertos no primeiro dia pós-operatório.

As pacientes serão aleatoriamente alocadas para os grupos:

- PO1 (n=100): o curativo será removido no primeiro dia pós-operatório;
- PO6 (n=100): a paciente será orientada a não molhar o curativo, e este será removido no sexto dia pós-operatório.
  1. **Procedimentos:**

Todas as pacientes serão internadas na véspera da cirurgia e tomarão um banho com solução degermante de clorexidina 4% no dia da cirurgia.^34^

- - 1. **Procedimento cirúrgico e curativo:**

As cirurgias serão realizadas sob anestesia geral, no centro cirúrgico do Hospital das Clínicas Samuel Libânio (HCSL), da Universidade do Vale do Sapucaí, Pouso Alegre-MG. A antissepsia do campo operatório será realizada com solução alcoólica de clorexidina 0,5%.^35^ A cirurgia oncológica será realizada pela equipe de Mastologia do HCSL, e a reconstrução mamária pela equipe do Setor de Cirurgia Plástica Mamária do HCSL. No caso de reconstrução mamária imediata, após o término da cirurgia oncológica será reaplicado antisséptico (clorexidina alcoólica 0,5%) no campo operatório e serão substituídos os campos e o instrumental cirúrgico para a realização do procedimento de reconstrução mamária. Todas as pacientes receberão antibioticoprofilaxia, conforme padronização do HCSL (cefazolina, 1g na indução anestésica e de 4/4h).

Ao final da operação, o sítio cirúrgico será limpo com solução salina estéril e será coletada uma amostra para cultura quantitativa. Em seguida, um curativo convencional com gazes será colocado: as feridas suturadas serão recobertas com quatro camadas de gazes de algodão estéreis, completamente recobertas e fixadas com fita microporosa. A equipe cirúrgica não saberá para qual grupo a paciente será alocada.

No momento da visita no primeiro dia pós-operatório, o cirurgião assistente abrirá um envelope opaco selado e numerado contendo a alocação da paciente. Pacientes que tiveram o curativo molhado ou substituído nessas primeiras 24 horas não serão incluídas.

As pacientes alocadas para o grupo PO1 terão seu curativo removido, e será feita uma segunda coleta de amostra para cultura. Estas pacientes serão orientadas a manter a ferida operatória descoberta, e a seguir sua rotina de higiene pessoal. As pacientes alocadas para o grupo PO6 serão orientadas a não molhar e não remover o curativo.

- - 1. **Coleta de amostras:**

Amostras para culturas serão obtidas na sala cirúrgica, antes do curativo, e imediatamente após a remoção do curativo. No grupo PO1, uma amostra adicional será coletada no sexto dia pós-operatório, por ocasião do retorno ambulatorial da paciente.

Serão passados, de forma padronizada, dois *swab* estéreis embebidos em solução salina sobre uma área padrão de 5cm por 10cm, determinada por um campo fenestrado de papel filtro estéril colocado sobre a ferida operatória. Estes *swabs* serão acondicionados em tubos estéreis contendo 1ml de solução salina e imediatamente conduzidos ao laboratório.

- - 1. **Método microbiológico:**

O mesmo técnico de laboratório processará todas as amostras. Serão utilizados métodos microbiológicos padrão para identificação de microoganismos.^36^ Aliquotas de 0,2ml da primeira amostra coletada serão semeadas em meios ágar hipertônico manitol, seletivo para *Staphylococcus sp*, ágar Sabouraud com cloranfenicol (0,05mg/ml), seletivo para fungos, ágar Teague EMB, seletivo para enterobactérias, e ágar sangue, para verificar a presença de colônias hemolíticas. As placas serão incubadas em ambiente aeróbico a 37°C. Após 48 horas a sete dias, a leitura do número de unidades formadoras de colônias (UFC) será realizada por um microbiologista. Estafilococos serão identificados como *Staphylococcus sp* coagulase-negativos ou *S. aureus* com base na coloração Gram, presença de hemólise e teste da coagulase. Aliquotas de 0,5ml da segunda amostra serão inoculadas em caldo tioglicolato e caldo glicosado e incubadas ambiente aeróbico a 37°C por 72 horas ou até cultura positiva, em um máximo de sete dias.

O mesmo microbiologista fará a leitura de todas as culturas. O técnico que processará as amostras e o microbiologista não saberão a qual grupo a amostra em questão pertence.

- - 1. **Infecção do sítio cirúrgico:**

O CDC considera infecção do sítio cirúrgico a que ocorre até 30 dias após a operação, quando não são utilizados implantes, ou até um ano após a cirurgia, nos casos em que houve implantes e a infecção aparenta estar relacionada ao procedimento cirúrgico.^37^

Assim, as pacientes serão sistematicamente seguidas quanto à ocorrência de infecção, uma vez por semana, durante os primeiros 30 dias, por um único cirurgião. Em todos os casos em que forem utilizados implantes, as pacientes serão reavaliadas, pelo mesmo cirurgião, um ano após o procedimento. Serão utilizadas as definições e classificações de infecção do sítio cirúrgico adotadas pelo CDC (Quadro 1).^37^

**Quadro 1** – Definições de infecção do sítio cirúrgico adotadas pelo CDC^37^

| **Incisional Superficial** | **Incisional Profunda** | **De órgão ou espaço** |
| --- | --- | --- |
| Envolve apenas pele ou tecido subcutâneo e apresenta pelo menos um dos seguintes itens:   - Secreção purulenta da porção superficial da incisão; - Isolamento de microorganismos em cultura de fluído ou tecido da porção superficial da incisão; - Pelo menos um dos seguintes sinais ou sintomas de infecção: dor, turgor, edema localizado, hiperemia ou calor, abertura deliberada da porção superficial da ferida pelo cirurgião, mesmo com cultura negativa; - Diagnóstico de infecção superficial pelo médico ou cirurgião assistente. | Envolve tecidos moles profundos (fáscia, músculo) e apresenta pelo menos um dos seguintes itens:   - Secreção purulenta de porção profunda da incisão, mas não envolvendo órgão ou espaço; - Porção profunda da incisão com deiscência espontânea ou deliberadamente aberta pelo cirurgião quando o paciente apresenta pelo menos um dos seguintes sinais ou sintomas: febre (>38°C), dor localizada ou turgor, mesmo com cultura negativa; - Abcesso ou outra evidência de infecção envolvendo porção profunda da incisão, encontrado em exame direto, durante reoperação ou em exame radiológico ou histopatológico; - Diagnóstico de infecção profunda pelo médico ou cirurgião assistente. | Envolve qualquer parte da anatomia (órgãos, espaços) e apresenta pelo menos um dos seguintes itens:   - Secreção purulenta oriunda de dreno colocado em órgão ou espaço; - Isolamento de microorganismos em cultura de fluído ou tecido coletados de órgão ou espaço; - Abcesso ou outra evidência de infecção envolvendo órgão ou espaço, encontrado em exame direto, durante reoperação ou em exame radiológico ou histopatológico; - Diagnóstico de infecção de órgão ou espaço pelo médico ou cirurgião assistente. |

- - 1. **Percepção das pacientes:**

Em seu retorno na segunda semana pós-operatória, as pacientes serão solicitadas a classificar sua percepção do tempo de manutenção do curativo (um dia ou seis dias), em relação à segurança, conforto e praticidade. Para isso, utilizarão uma escala de cinco pontos tipo Likert (excelente, muito bom, bom, regular, ruim).

Além disso, serão solicitadas a responder à seguinte questão: “Independente do tempo que seu curativo permaneceu, se você pudesse escolher, preferiria ficar com o curativo um dia ou seis dias?”

- - 1. **Análise estatística:**

Os dados serão tabulados e submetidos à análise estatística. Para análise dos resultados, serão utilizados testes paramétricos ou não paramétricos, dependendo da natureza das variáveis ou da variabilidade dos valores encontrados.

1. **REFERÊNCIAS**
2. Gravante G, Caruso R, Araco A, Cervelli V. Infections after plastic procedures: incidences, etiologies risk factors, and antibiotic prophylaxis. *Aesth Plast Surg.* 2008; 32: 243-251
3. Andenaes K, Amland PF, Lingaas E, Abyholm F, Samdal F, Giercksky KE. A prospective, randomized surveillance study of postoperative wound infections after plastic surgery: a study of incidence and surveillance methods. *Plast Reconstr Surg.* 1995; 96: 948-956
4. Oliveira AC, Braz NJ, Ribeiro MM. Incidência da infecção do sítio cirúrgico em um hospital universitário. Cienc Cuid Saude. 2007; 6: 486-93
5. Nichols RL. Preventing surgical site infections: a surgeon's perspective. Emerg Infect Dis. 2001; 7: 220-4.
6. Perotti J A, Castor SA, Perez PC, Zins JE. Antibiotic use in aesthetic surgery: a national survey and literature review. Plast Reconstr Surg 2002; 109: 1685-93
7. Olsen MA, Chu-Ongsakul S, Brandt KE, Dietz JR, Mayfield J, Fraser V. Hospital-associated costs due to surgical site infection after breast surgery. *Arch Surg.* 2008; 143: 53-60
8. Tejirian T, DiFronzo LA, Haigh PI. Antibiotic prophylaxis for preventing wound infection after breast surgery: a systematic review and metaanalysis. *J Am Coll Surg.* 2006; 203: 729-734
9. Hall JC, Willsher PC, Hall JL. Randomized clinical trial of single-dose antibiotic prophylaxis for non-reconstructive breast surgery. *Br J Surg.* 2006; 93: 1342-1346
10. Tran CL, Langer S, Broderick-Villa G, DiFronzo LA. Does reoperation predispose to postoperative wound infection in women undergoing operation for breast cancer? Am Surg. 2003; 69: 852-6
11. Bertin ML, Crowe J, Gordon SM. Determinants of surgical site infection after breast surgery. Am J Infect Control. 1998; 26: 61-5
12. Chen J, Gutkin Z, Bawnik J. Postoperative infections in breast surgery. J Hosp Infect. 1991; 17: 61-5
13. Olsen MA, Lefta M, Dietz JR, Brandt KE, Aft R, Matthews R, et al. Risk factors for surgical site infection after major breast operation. *J Am Coll Surg.* 2008; 207: 326-335
14. Alderman AK, Wilkins EG, Kim HM, Lowery JC. Complications in postmastectomy breast reconstruction: two-year results of the Michigan Breast Reconstruction Outcome Study. Plast Reconstr Surg. 2002; 109: 2265-74
15. Nahabedian MY, Tsangaris T, Momen B,Manson PN. Infectious complications following breast reconstruction with expanders and implants. Plast Reconstr Surg. 2003; 112: 467-76
16. Landes G, Harris PG, Lemaine V, Perreault I, Sampalis JS, Brutus JP, et al. Prevention of surgical site infection and appropriateness of antibiotic prescribing habits in plastic surgery. *J Plast Recontr Aesth Surg.* 2008; 61: 1347-1356
17. Sørensen LT, Hørby J, Friis E, Pilsgaard B, Jørgensen T. Smoking as a risk factor for wound healing and infection in breast cancer surgery. *ESJO.* 2002; 28: 815-820
18. Anderson DJ, Kaye KS. Staphylococcal surgical site infections. *Infect Dis Clin N Am.* 2009; 23: 53-72
19. Mangram AJ, Horan TC, Pearson ML, Silver LC, Jarvis WR, The Hospital Infection Control Practices Advisory Committee. Guideline for prevention of surgical site infection. *Am J Infect Control*. 1999; 27: 97-134
20. Segers P, de Jong AP, Spanjaard L, Ubbink DT, de Mol BAJM. Randomized clinical trial comparing two options for postoperative incisional care to prevent poststernotomy surgical site infections. *Wound Rep Reg.* 2007; 15: 192-196
21. Lionelli GT, Lawrence WT. Wound dressings. *Surg Clin N Am.* 2003; 83: 617-638
22. Michie DD, Hugill JV. Influence of occlusive and impregnated gauze dressings on incisional healing: a prospective, randomized, controlled study. *Ann Plast Surg.* 1994; 32: 57-64
23. Chrintz H, Cordtz TO, Harreby JS, Waaddegaard P, Larsen SO. Need for surgical wound dressing. *Br J Surg.* 1989; 76: 204-205
24. Cho CY, Lo JS. Dressing the part. *Dermatol Clin.* 1998; 16: 25-47
25. Al-Benna S. An easy and comfortable way of maintaining dressings in breast surgery - reply.  *Plast Reconstr Surg.* 2008; 121: 680-681
26. Benito P, De Juan A, Cano M, Elena E. An easy and comfortable way of maintaining dressings in breast surgery. *Plast Reconstr Surg.* 2008; 121: 680-681
27. Paddle-Ledinek JE, Nasa Z, Cleland H. Effect of different wound dressings on cell viability and proliferation. *Plast Reconstr Surg.* 2006; 117(Suppl.): 110S-118S
28. Wynne R, Botti M, Stedman H, Holsworth L, Harinos M, Flavell O, et al. Effect of three wound dressings on infection, healing comfort, and cost in patients with sternotomy wounds: a randomized trial. *Chest.* 2004; 125: 43-49
29. Sticha RS, Swiriduk D, Wertheimer SJ. Prospective analysis of postoperative wound infections using an early exposure method of wound care. *J Foot Ankle Surg.* 1998; 37: 286-291
30. Heal C, Buettner P, Raasch B, Browning S, Graham D, Bidgood R, et al. Can sutures get wet? Prospective randomized controlled trial of wound management in general practice. *BMJ.* 2006; 332: 1053-1056
31. Holm C, Petersen JS, Gronboek F, Gottrup F. Effects of occlusive and conventional gauze dressings on incisional healing after abdominal operations. *Eur J Surg.* 1998; 164: 179-183
32. Thomas DW, Hill CM, Lewis MAO, Stephens P, Walker R, Weth AVD. Randomized clinical trial of the effect of semi-occlusive dressings on the microflora and clinical outcome of acute facial wounds. Wound Rep Reg. 2000; 8: 258-63
33. Rosenfeldt FL, Negri J, Holdaway D, Davis BB, Mack J, Grigg MJ, et al. Occlusive wrap dressing reduces infection rate in saphenous vein harvest site. *Ann Thorac Surg.* 2003; 75: 101-105
34. Alvarez JM. Use of an occlusive dressing for 2 weeks reduces the incidence of esternal wound infections. *ANZ J Surg.* 2005; 75: 179-180
35. Veiga DF, Damasceno CAV, Veiga-Filho J, Figueiras RG, Vieira RB, Garcia ES, et al. Randomized controlled trial on the effectiveness of chlorhexidine showers before elective plastic surgical procedures. *Infect Control Hosp Epidemiol.* 2009; 30: 77-79
36. Veiga DF, Damasceno CAV, Veiga-Filho J, Figueiras RG, Vieira RB, Florenzano FH, et al. Povidone-iodine (PVP-I) versus chlorhexidine in antisepsis before elective plastic surgery procedures: randomized controlled trial. *Plast Reconstr Surg.* 2008; 122: 170e-171e
37. Trabulsi LR, Alterthum F. *Microbiologia*. 4th ed. São Paulo: Atheneu; 2005
38. Horan TC, Gaynes RPG, Martone WJ, Jarvis WR, Emori TG. CDC definitions of nosocomial surgical site infections, 1992: a modification of CDC definitions of surgical wound infections*. Infect Control Hosp Epidemiol.* 1992; 13: 606-608
39. Jones VJ. The use of gauze: will it ever change? *Int Wound J*. 2006; 3: 79-86
40. **ANEXOS**

## Termo de Consentimento Livre e Esclarecido

**Responsável:** Dra. Daniela Francescato Veiga

**Título:** TEMPO DE MANUTENÇÃO DO CURATIVO APÓS RECONSTRUÇÃO MAMÁRIA: INFLUÊNCIA NA COLONIZAÇÃO CUTÂNEA E OCORRÊNCIA DE INFECÇÃO DO SÍTIO CIRÚRGICO

_____________________________________________________________________________

Eu compreendo que fui convidada para participar como voluntária nesta pesquisa. O objetivo deste estudo é avaliar a influência do tempo de permanência do curativo convencional com gazes, após reconstrução mamária, na colonização cutânea e na taxa de infecção do sítio cirúrgico. Não existe um consenso sobre isso, em alguns hospitais é rotina deixar o curativo por vários dias e em outros a rotina é retirar o curativo no primeiro dia após a cirurgia. Não se sabe qual das duas formas é a melhor. Para isso, as pacientes que participarão do estudo poderão ficar com o curativo por um dia (grupo PO1) ou por seis dias (grupo PO6). Será feito um sorteio para decidir o número de dias que o seu curativo vai ficar.

**Riscos para a paciente:** Riscos habituais a qualquer procedimento cirúrgico e anestésico, desde hemorragias, infecção, deiscências de sutura, cicatriz hipertrófica, bem como dor pós-operatória.

**Direito de privacidade:**

Estou ciente de que a minha identidade será preservada e que as informações obtidas com a pesquisa serão divulgadas, de forma que os resultados não poderão ser relacionados à minha pessoa.

**Declaração de danos:**

Caso ocorra algum dano á minha pessoa, resultante diretamente da minha participação nesta pesquisa, o pesquisador e a Instituição serão os responsáveis. Terei também, gratuitamente, todo o tratamento para recuperar algum dano causado pela pesquisa.

**______________________________________________________________________**

**Recusa ou retirada:**

Eu compreendo que minha participação é voluntária e eu posso recusar e retirar o meu consentimento a qualquer momento, sem que isso prejudique meu cuidado atual ou futuro nesta Instituição.

Confirmo que a Dra. Daniela F. Veiga ou um de seus assistentes explicaram-me os objetivos desta pesquisa e os procedimentos a que serei submetida (realização de procedimento cirúrgico) e que li e compreendi este formulário de consentimento. Portanto, concordo em participar desta pesquisa, e uma cópia deste termo ficará em meu poder.

**Pouso Alegre, ____/____/____**

**_______________________________ ______________________________**

**Paciente ou Responsável**  **Pesquisador**

PACIENTE Nº________

Nome: _____________________________________________ Idade:_____ Cor:_____

**Dados da Cirurgia:**

Alocado para o grupo: ( ) PO1 ( ) PO6

Tipo cirurgia: ______________________________________ Data: ____ / ___ / ____

Tipo anestesia: ______________________ Tempo de cirurgia: ___________________

Intercorrências:_________________________________________________________

Antibiótico: ( ) não ( ) sim

Qual:_________________________ Dose/tempo: _____________________________

**Resultados Microbiológicos:**

Antes do curativo:

| A. Manitol | Manitol + | Coagulase | Gram | A. Saboraud | A. Teague | A. Sangue | Hemólise |
| --- | --- | --- | --- | --- | --- | --- | --- |
|  |  |  |  |  |  |  |  |

PO 1:

| A. Manitol | Manitol + | Coagulase | Gram | A. Saboraud | A. Teague | A. Sangue | Hemólise |
| --- | --- | --- | --- | --- | --- | --- | --- |
|  |  |  |  |  |  |  |  |

PO 6:

| A. Manitol | Manitol + | Coagulase | Gram | A. Saboraud | A. Teague | A. Sangue | Hemólise |
| --- | --- | --- | --- | --- | --- | --- | --- |
|  |  |  |  |  |  |  |  |

**Dados do seguimento pós-operatório:**

**Semana 1:** ( ) febre ( )secreções ( ) sinais flogísticos? Quais:_________________________________________________________________Obs: __________________________________________________________________

**Semana 2:** ( ) febre ( )secreções ( ) sinais flogísticos? Quais:_________________________________________________________________Obs: __________________________________________________________________

**Semana 3:** ( ) febre ( )secreções ( ) sinais flogísticos? Quais:_________________________________________________________________Obs: __________________________________________________________________

**Semana 4:** ( ) febre ( )secreções ( ) sinais flogísticos? Quais:_________________________________________________________________Obs: __________________________________________________________________

**Um ano:** ( ) febre ( )secreções ( ) sinais flogísticos? Quais:_________________________________________________________________Obs: __________________________________________________________________

Como você avaliaria o tempo que foi mantido o curativo que foi realizado após sua cirurgia das mamas, em relação a:

1. **Segurança**

( ) Excelente ( ) Muito bom ( ) Bom ( ) Regular ( ) Ruim

1. **Conforto**

( ) Excelente ( ) Muito bom ( ) Bom ( ) Regular ( ) Ruim

1. **Praticidade**

( ) Excelente ( ) Muito bom ( ) Bom ( ) Regular ( ) Ruim

1. Independente de quantos dias você ficou com o curativo, se você pudesse escolher, você ficaria com o curativo por:

( ) 1 dia ( ) 6 dias


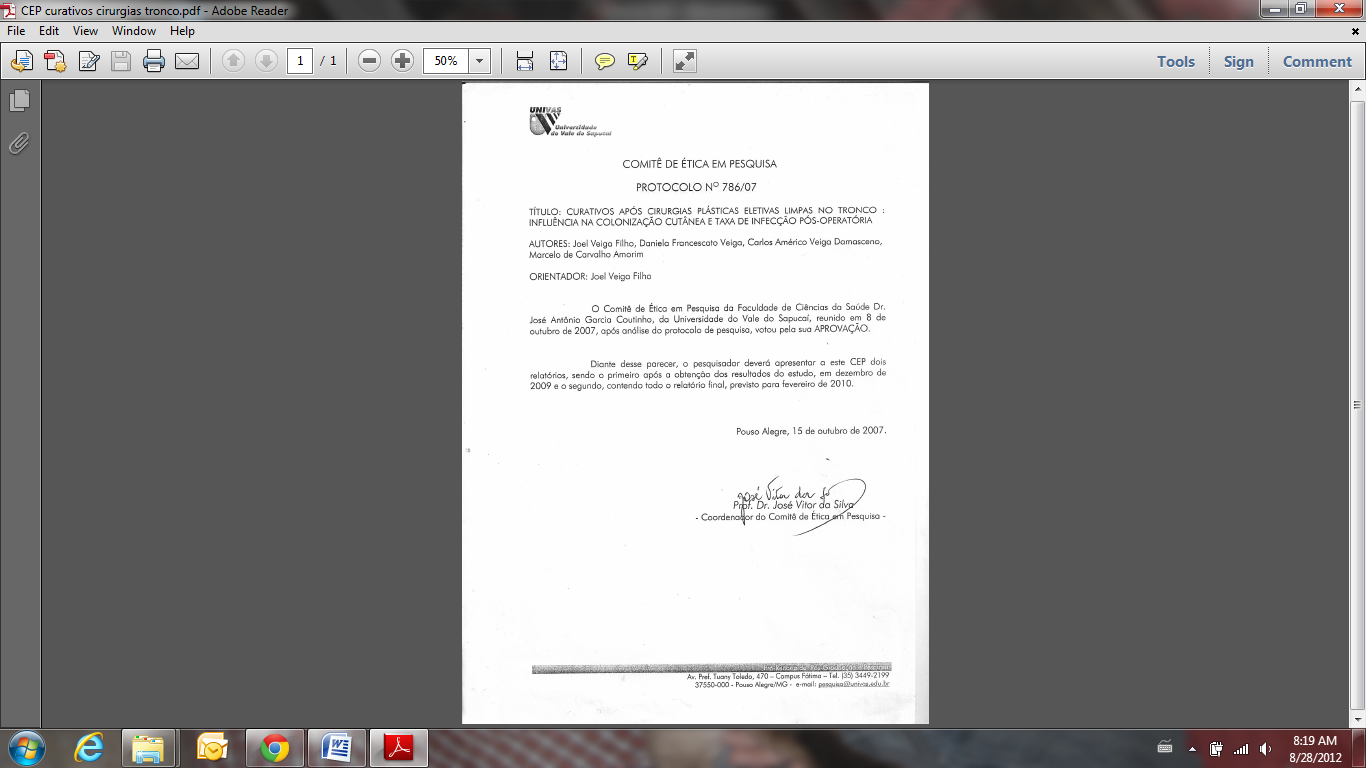


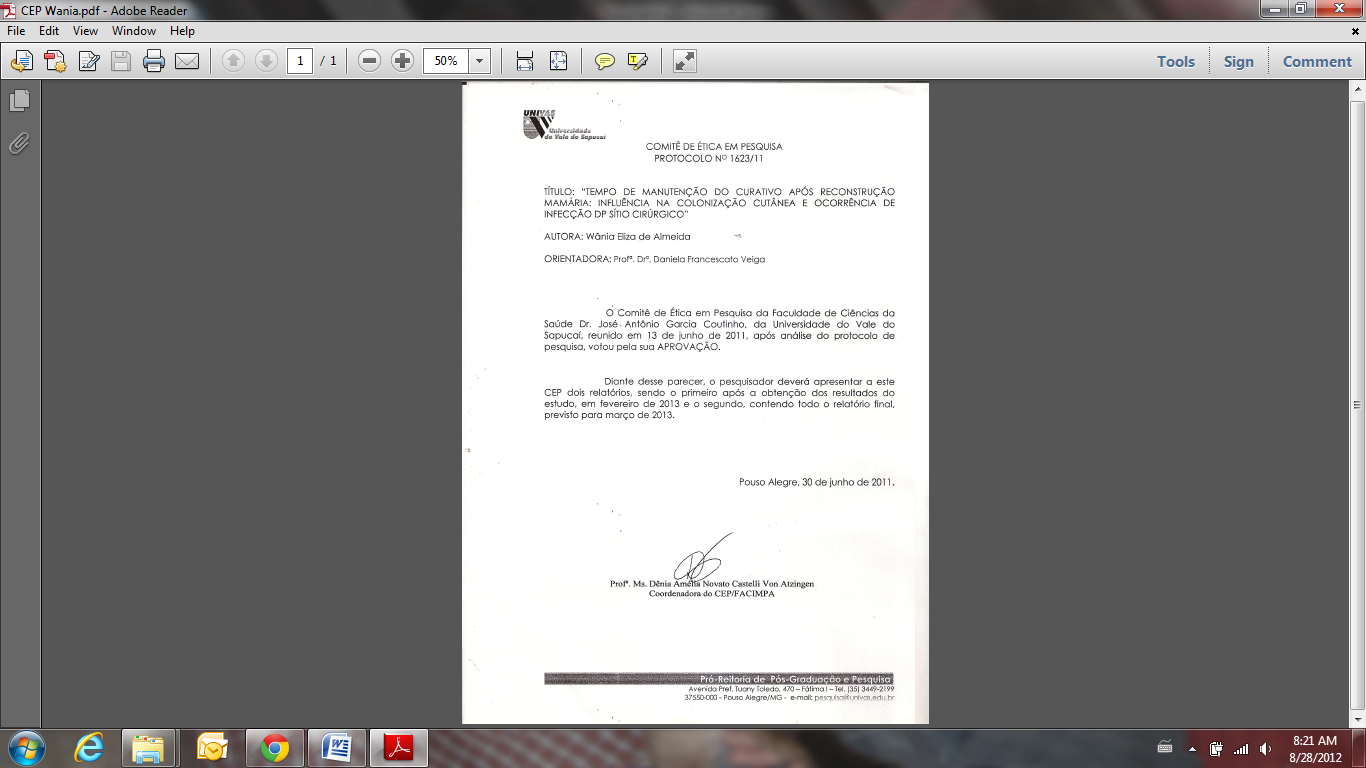

Supplement: S1 File — (DOC) [file pone.0166356.s001.doc]
