# Supplementary material for: Dressing Wear Time after Breast Reconstruction: A Randomized Clinical Trial
Source: PLoS One. 2016 Dec 2;11(12):e0166356. doi: 10.1371/journal.pone.0166356 (PMC5135046; doi:10.1371/journal.pone.0166356)
Supplement: S2 File — (DOC) [file pone.0166356.s002.doc]

**DRESSING WEAR TIME AFTER BREAST RECONSTRUCTION: INFLUENCE ON SKIN COLONIZATION AND SURGICAL SITE INFECTION RATES**

1. **ABSTRACT**

**Setting:** The study will be developed at the Plastic Surgery Graduate Program - Universidade Federal de São Paulo – UNIFESP and at the Universidade do Vale do Sapucaí – UNIVÁS

**Background:** Surgical site infection is a relevant problem, with many issues to be elucidate, and remains a major cause of morbidity among surgical patients. Despite postoperative wound management is part of the surgeon routine, the evidence to support dressing standards is empiric and scarce.

**Aims:** To assess the influence of dressing wear time on surgical site infection rates and skin colonization. Patients’ perception at self-assessment will also be analyzed.

**Methods:** This is a two-arm randomized controlled trial. Two hundred breast cancer patients undergoing immediate or delayed breast reconstruction will be prospectively enrolled. Patients will be randomly allocated to group I (dressing removed on postoperative day 1) or group II (dressing removed on postoperative day 6). Skin colonization will be assessed by culture of samples collected before dressing and immediately after removing dressing. Surgical site infections will be defined by standard criteria from the Centers for Disease Control and Prevention (CDC). Patients will be assessed weekly, for 30 days, and patients who received an implant will be reassessed after one year. Patients will score dressing wear time in regard to safety, comfort and convenience.

**Key-words:** breast cancer; breast reconstruction; postoperative care; dressings; surgical wound infection; bacterial growth.

1. **INTRODUCTION**

Surgical site infection (SSI) is a relevant problem in surgery practice, with issues that still need to be clarified, and is a major cause of morbidity among surgical patients.^1,2^ In Brazil, the estimated incidence of SSI is about 11% of all performed operations.^3^ At United States of America, SSI is responsible for one quarter of the hospital infections, leading to increased time of hospitalization and higher costs.^4^

The infection risk for clean wounds is estimated to be 1 to 2%.^5^ SSI rates following breast surgery seem to be much higher than what is expected for clean surgical procedures.^6-8^ The incidence of SSI after mastectomy varies from 2.8% to 25% in literature,^6,9-11^ and SSI rates after breast reconstructive procedures ranges from 6.3% to 28%.^6,12-16^ To understand the risk factors for breast surgery is essential to the development of prevention strategies.^12^

Risk factors for SSI are usually classified into three categories: patient-related (preoperative), procedure-related (perioperative) and postoperative related.^17^ Factors related to patient include age, obesity, tobacco use, comorbidities, use of immunosuppressive medications. Factors related to the procedure include type and duration of the operation, hypoxia, traffic in the operating room and operating room parameters, among others. ^17,18^ One of the major risk factors in the postoperative period is wound management. ^17-19^

Postoperative wound care is an ancient practice, with recorded evidence dating from 4000 years.^20^ Purposes for wound dressing include protection of the wound from trauma and contamination, absorption of wound exudates and compression to minimize edema and obliterate dead space. Besides, dressing provides a better appearance of the operated site, leading to patient's psychological well-being.^21^ The ideal dressing should protect wounds from injury and contamination; absorb wound secretion; and provide compression to minimize edema and obliterate dead space.^20,22,23^

The search for an ideal postsurgical breast dressing has led to the development of several different materials and application techniques. ^24,25^ Despite the abundance of wound dressing products available nowadays, there is little empiric evidence to guide product choice for site-specific incisional wounds, including breast surgical wounds. ^19,21-23,26,27^

The ideal dressing wear time is controversial in literature. Some authors recommend the early exposure of the surgical wound, to allow easy wound inspection without inconvenience to the patient, to release patient for his/her routine personal care and to decrease costs. ^22,28,29^ Chrintz *et al.*, in a randomized trial with 1202 patients, found no differences on SSI rates when dressing was removed on the first postoperative day, compared to dressing kept in place until removal of sutures.^22^

Other authors recommend to keep dressing placed at operating room, under ideal antisepsis conditions, until sutures are removed, if the dressing is dry. ^28,30-33^ Alvarez demonstrou que o uso de curativo oclusivo por duas semanas, após esternotomia em cirurgias cardíacas, diminuiu a incidência de infecção pós-operatória.^33^ Rosenfeldt *et al.* demonstraram que o curativo oclusivo reduziu a taxa de infecção pós-operatória em cirurgias no membro inferior.^32^

The management of surgical wounds should involve the principle of minimizing harm, and patient preference and tolerance must be considered.^26^ Chrintz et al. pointed that omitting a dressing after the first 24 postoperative hours could be convenient for patients, allowing them to carry out their personal hygiene more easily.^22^ On the other hand, other authors observed that dressings are comforting to patients by masking the scars.^23,27^

Despite the management of the surgical wound is part of the surgeons routine, the conduct regarding wound management is based on scarce scientific evidence, and dressing wear time is based on tradition, not on evidences. ^28,29^

The Centers for Disease Control and Prevention' s (CDC) guidelines instruct that wounds that are closed primarily should be covered with a sterile dressing for 24-48 hours.^18^ There is neither recommendation for covering a primarily closed incision beyond 48 hours nor for the appropriate time to let patients to shower. These remain unsolved issues.^18^

Thus, this randomized controlled trial was designed to assess the influence of dressing wear time after breast reconstruction on SSI rates, skin colonization and patients’ perceptions, which always must be considered.

1. **OBJECTIVES**

- 1. **Primary aim:**
- To assess the influence of dressing wear time after breast reconstruction on SSI rates.
  1. **Secondary aims:**
- To assess the influence of dressing wear time on skin colonization;
- To assess patients’ perceptions on dressing wear time.

1. **MÉTODOS**
   1. **Design:**

This is a primary, clinical, prospective, randomized, controlled interventional, analytic trial.

- 1. **Participants:**
     1. **Sample size:**

Considering that SSI rates following breast reconstruction range, in literature, from 2% a 28%,^6,12-16^ and considering clinically relevant a 10% difference in SSI rate, the calculated sample size was 100 patients per arm, with a significance level of 5% and a power of 80%.

- - 1. **Recruitment:**

A total of 200 breast cancer patients, candidates to immediate breast reconstruction or previously submitted to surgical treatment of breast cancer will be recruited from the Mastology and Breast Plastic Surgery ambulatory units of the Hospital das Clínicas Samuel Libânio (HCSL), of Universidade do Vale do Sapucaí, Pouso Alegre – MG. Patients who meet the eligibility criteria will be invited and only participants who agree to provide written informed consent will be included in the study.

The following eligibility criteria will be considered:

Inclusion criteria:

- Female, between 18 and 70 years-old, with no restrictions regarding ethnicity, education or social level;
- Patients who had undergone surgical treatment of breast cancer, candidates to breast reconstruction.

Exclusion criteria:

- Co morbidities which are usual contraindications for breast reconstruction procedures;
- heavy smoking;
- Body mass index (BMI) above 35Kg/m^2^;
- Patients who had dressing wet in the first 24 hours after operation, requiring change.
  - 1. **Allocation:**

The allocation will be determined by a computer-generated sequence (Bioestat 5.0, Instituto de Desenvolvimento Sustentável Mamirauá, Belém, PA, Brazil). A sealed opaque envelope with patient’s number in the study will be opened in the first postoperative day to reveal the allocation of the patient.

Patients will be randomly assigned to the groups: I (n=100), which will have dressings removed in the first postoperative day or to group II (n=100), whose dressings will be removed in the sixth postoperative day. As pacientes serão aleatoriamente alocadas para os grupos:

- PO1 (n=100): dressings removed in the first postoperative day;
- PO6 (n=100): dressings removed in the sixth postoperative day.
  1. **Procedures:**

Patients will be admitted the day before the operation, and they will take a shower with liquid detergent-based chlorhexidine 4% prior to the operation.^34^

- - 1. **Surgical procedures and dressings:**

Operations will be performed under general anesthesia, at the surgical center of the Hospital das Clínicas Samuel Libânio (HCSL), Universidade do Vale do Sapucaí, Pouso Alegre-MG. An alcoholic solution of chlorhexidine 0.5% will be used for the antisepsis of the surgical site antissepsia.^35^

The oncologic operation will be performed by the Mastology teaam of the HCSL, and breast reconstruction by the breast plastic surgery team of the HCSL. Whenever an immediate breast reconstruction is performed, at the end of the oncologic the antisseptic solution (alcoholic chlorhexidine 0.5%) will be reapplied to the surgical site. All patients will receive prophylactic antibiotics (cephazolin, 1g at the beginning of anesthesia and each four hours).

At the end of the operation, surgical site will be cleansed with sterile physiological saline and samples for skin culture will be obtained. Then, a conventional gauze and tape dressing will be placed: sutured wounds will be covered with four layers of dry sterile cotton gauze, completely covered and fixed in place by a micropore tape. Surgical team will not be aware of which group the patient will be allocated.

At the first postoperative day, the assistant surgeon will open a sealed, opaque and numbered envelope to reveal the allocation of the patient. Patients who had dressing wet or changed in the first 24 hours will not be included.

Patients allocated to group PO1 will have their dressings removed, and a second sample for culture will be collected. These patients will be instructed to keep wounds uncovered and follow their usual personal hygiene routine. Patients allocated to group PO6 will be instructed not to wet or to remove the dressing.

- - 1. **Samples for cultures:**

Samples for cultures will be obtained in the operating room, immediately before placing the dressing, as well as immediately after the removal of dressing. In group PO1, an additional sample will be obtained on the sixth postoperative day, at the ambulatory unit.

A standard 5 by 10cm area (determined by a sterile pattern) over the surgical wound will be swabbed with sterile cotton swabs pre moistened with sterile saline. These swabs will be placed in a sterile container with 1.0 ml of saline and immediately conducted to the laboratory.

- - 1. **Microbiological methods:**

The same laboratory technician will process all the samples. Standard microbiologic methods and criteria will be used to identify microorganisms.^36^ Aliquots of 0.2 ml of the sample will be plated on hypertonic manitol (HM) agar, selective for staphylococci, on blood agar, to identify hemolytic colonies, on Sabouraud agar with chloramphenicol (0.05mg/ml), selective for fungi and yeasts, and on eosin-methylene blue (EMB) agar, selective for enterobacteria. Plates will be incubated aerobically at 37°C. After 48 hours to seven days, plates will be examined and colony forming unities (CFU) will be counted by a microbiologist. Staphylococci will be identified as coagulase-negative *Staphylococcus* sp. or *S. aureus* on the basis of Gram stain, presence of hemolysis and coagulase testing. The same microbiologist will assess all the plates. Both the laboratory technician and the microbiologist will be blinded. Aliquots of 0.5 ml of the second sample were inoculated into thioglycolate broth and glucose broth and incubated aerobically at 37°C for 72h or until positive, at a maximum of seven days. The same microbiologist will assess all the cultures. Both the laboratory technician and the microbiologist will be blinded.

- - 1. **Surgical Site Infection (SSI):**

The CDC considers SSI the infection that occurs within 30 days after the operative procedure if no implant is left in place or within one year if implant is in place and the infection appears to be related to the operative procedure.^37^

Thus, patients will be systematically followed-up once a week for 30 days, regarding to postoperative infection, by a single surgeon. Patients who receive an implant will have an additional assessment, by the same surgeon, one year after the operation. The CDC definitions and classifications of SSI will be considered (table 1).^37^

**Table 1** – CDC definitions of SS^37^

| **Superficial Incisional SSI** | **Deep Incisional SSI** | **Organ/Space SSI** |
| --- | --- | --- |
| Involves only skin or subcutaneous tissue and meets at least one of the following:   - Purulent drainage from the superficial incision; - Organisms isolated from an aseptically obtained culture of fluid or tissue from the superficial incision; - At least one of the following signs or symptoms of infection: pain or tenderness, localized swelling, redness or heat, and the superficial incision is deliberately opened by surgeon unless the incision is culture-negative; - Diagnosis of superficial incisional SSI by the surgeon or attending physician. | Involves deep soft tissues (fascial and muscle layers) and meets at least one of the following:   - Purulent drainage from the deep incision but not from the organ/space component of the surgical site; - A deep incision that spontaneously dehisces or is deliberately opened by a surgeon when the patient has at least one of the following signs or symptoms: fever (>38°C), localized pain or tenderness, unless the incision is culture-negative; - An abscess or other evidence of infection involving the deep incision is found on direct examination, during reoperation, or by histopathologic or radiologic examination; - Diagnosis of deep incisional SSI by the surgeon or attending physician. | Involves any part of the anatomy (organs or spaces) and meets at least one of the following:   - Purulent drainage from a drain that is placed through a stab wound into the organ/space; - Organisms isolated from an aseptically obtained culture of fluid or tissue in the organ/space; - An abscess or other evidence of infection involving the organ/space that is found on direct examination, during reoperation, or by histopathologic or radiologic examination; - Diagnosis of an organ/space SSI by the surgeon or attending physician. |

- - 1. **Patients' perceptions:**

On their return in the second week after operation, patients will be asked to rate their dressing wear time (one day or six days) in regard to safety, comfort and convenience, by the use of a Likert 5-point rating scale (excellent, very good, good, fair, poor).

Besides, they will be asked to answer the following question: “Regardless of how many time your dressing was left in place, if you had the choice, would you prefer to keep the dressing for one day or for six days?”

- - 1. **Statistical analysis:**

The data will be tabulated and analyzed statistically. For data analysis, parametric or non-parametric tests will be used, depending on the nature of the variables or the variability of the values found.

1. **REFERENCES**
2. Gravante G, Caruso R, Araco A, Cervelli V. Infections after plastic procedures: incidences, etiologies risk factors, and antibiotic prophylaxis. *Aesth Plast Surg.* 2008; 32: 243-251
3. Andenaes K, Amland PF, Lingaas E, Abyholm F, Samdal F, Giercksky KE. A prospective, randomized surveillance study of postoperative wound infections after plastic surgery: a study of incidence and surveillance methods. *Plast Reconstr Surg.* 1995; 96: 948-956
4. Oliveira AC, Braz NJ, Ribeiro MM. Incidência da infecção do sítio cirúrgico em um hospital universitário. Cienc Cuid Saude. 2007; 6: 486-93
5. Nichols RL. Preventing surgical site infections: a surgeon's perspective. Emerg Infect Dis. 2001; 7: 220-4.
6. Perotti J A, Castor SA, Perez PC, Zins JE. Antibiotic use in aesthetic surgery: a national survey and literature review. Plast Reconstr Surg 2002; 109: 1685-93
7. Olsen MA, Chu-Ongsakul S, Brandt KE, Dietz JR, Mayfield J, Fraser V. Hospital-associated costs due to surgical site infection after breast surgery. *Arch Surg.* 2008; 143: 53-60
8. Tejirian T, DiFronzo LA, Haigh PI. Antibiotic prophylaxis for preventing wound infection after breast surgery: a systematic review and metaanalysis. *J Am Coll Surg.* 2006; 203: 729-734
9. Hall JC, Willsher PC, Hall JL. Randomized clinical trial of single-dose antibiotic prophylaxis for non-reconstructive breast surgery. *Br J Surg.* 2006; 93: 1342-1346
10. Tran CL, Langer S, Broderick-Villa G, DiFronzo LA. Does reoperation predispose to postoperative wound infection in women undergoing operation for breast cancer? Am Surg. 2003; 69: 852-6
11. Bertin ML, Crowe J, Gordon SM. Determinants of surgical site infection after breast surgery. Am J Infect Control. 1998; 26: 61-5
12. Chen J, Gutkin Z, Bawnik J. Postoperative infections in breast surgery. J Hosp Infect. 1991; 17: 61-5
13. Olsen MA, Lefta M, Dietz JR, Brandt KE, Aft R, Matthews R, et al. Risk factors for surgical site infection after major breast operation. *J Am Coll Surg.* 2008; 207: 326-335
14. Alderman AK, Wilkins EG, Kim HM, Lowery JC. Complications in postmastectomy breast reconstruction: two-year results of the Michigan Breast Reconstruction Outcome Study. Plast Reconstr Surg. 2002; 109: 2265-74
15. Nahabedian MY, Tsangaris T, Momen B,Manson PN. Infectious complications following breast reconstruction with expanders and implants. Plast Reconstr Surg. 2003; 112: 467-76
16. Landes G, Harris PG, Lemaine V, Perreault I, Sampalis JS, Brutus JP, et al. Prevention of surgical site infection and appropriateness of antibiotic prescribing habits in plastic surgery. *J Plast Recontr Aesth Surg.* 2008; 61: 1347-1356
17. Sørensen LT, Hørby J, Friis E, Pilsgaard B, Jørgensen T. Smoking as a risk factor for wound healing and infection in breast cancer surgery. *ESJO.* 2002; 28: 815-820
18. Anderson DJ, Kaye KS. Staphylococcal surgical site infections. *Infect Dis Clin N Am.* 2009; 23: 53-72
19. Mangram AJ, Horan TC, Pearson ML, Silver LC, Jarvis WR, The Hospital Infection Control Practices Advisory Committee. Guideline for prevention of surgical site infection. *Am J Infect Control*. 1999; 27: 97-134
20. Segers P, de Jong AP, Spanjaard L, Ubbink DT, de Mol BAJM. Randomized clinical trial comparing two options for postoperative incisional care to prevent poststernotomy surgical site infections. *Wound Rep Reg.* 2007; 15: 192-196
21. Lionelli GT, Lawrence WT. Wound dressings. *Surg Clin N Am.* 2003; 83: 617-638
22. Michie DD, Hugill JV. Influence of occlusive and impregnated gauze dressings on incisional healing: a prospective, randomized, controlled study. *Ann Plast Surg.* 1994; 32: 57-64
23. Chrintz H, Cordtz TO, Harreby JS, Waaddegaard P, Larsen SO. Need for surgical wound dressing. *Br J Surg.* 1989; 76: 204-205
24. Cho CY, Lo JS. Dressing the part. *Dermatol Clin.* 1998; 16: 25-47
25. Al-Benna S. An easy and comfortable way of maintaining dressings in breast surgery - reply.  *Plast Reconstr Surg.* 2008; 121: 680-681
26. Benito P, De Juan A, Cano M, Elena E. An easy and comfortable way of maintaining dressings in breast surgery. *Plast Reconstr Surg.* 2008; 121: 680-681
27. Paddle-Ledinek JE, Nasa Z, Cleland H. Effect of different wound dressings on cell viability and proliferation. *Plast Reconstr Surg.* 2006; 117(Suppl.): 110S-118S
28. Wynne R, Botti M, Stedman H, Holsworth L, Harinos M, Flavell O, et al. Effect of three wound dressings on infection, healing comfort, and cost in patients with sternotomy wounds: a randomized trial. *Chest.* 2004; 125: 43-49
29. Sticha RS, Swiriduk D, Wertheimer SJ. Prospective analysis of postoperative wound infections using an early exposure method of wound care. *J Foot Ankle Surg.* 1998; 37: 286-291
30. Heal C, Buettner P, Raasch B, Browning S, Graham D, Bidgood R, et al. Can sutures get wet? Prospective randomized controlled trial of wound management in general practice. *BMJ.* 2006; 332: 1053-1056
31. Holm C, Petersen JS, Gronboek F, Gottrup F. Effects of occlusive and conventional gauze dressings on incisional healing after abdominal operations. *Eur J Surg.* 1998; 164: 179-183
32. Thomas DW, Hill CM, Lewis MAO, Stephens P, Walker R, Weth AVD. Randomized clinical trial of the effect of semi-occlusive dressings on the microflora and clinical outcome of acute facial wounds. Wound Rep Reg. 2000; 8: 258-63
33. Rosenfeldt FL, Negri J, Holdaway D, Davis BB, Mack J, Grigg MJ, et al. Occlusive wrap dressing reduces infection rate in saphenous vein harvest site. *Ann Thorac Surg.* 2003; 75: 101-105
34. Alvarez JM. Use of an occlusive dressing for 2 weeks reduces the incidence of esternal wound infections. *ANZ J Surg.* 2005; 75: 179-180
35. Veiga DF, Damasceno CAV, Veiga-Filho J, Figueiras RG, Vieira RB, Garcia ES, et al. Randomized controlled trial on the effectiveness of chlorhexidine showers before elective plastic surgical procedures. *Infect Control Hosp Epidemiol.* 2009; 30: 77-79
36. Veiga DF, Damasceno CAV, Veiga-Filho J, Figueiras RG, Vieira RB, Florenzano FH, et al. Povidone-iodine (PVP-I) versus chlorhexidine in antisepsis before elective plastic surgery procedures: randomized controlled trial. *Plast Reconstr Surg.* 2008; 122: 170e-171e
37. Trabulsi LR, Alterthum F. *Microbiologia*. 4th ed. São Paulo: Atheneu; 2005
38. Horan TC, Gaynes RPG, Martone WJ, Jarvis WR, Emori TG. CDC definitions of nosocomial surgical site infections, 1992: a modification of CDC definitions of surgical wound infections*. Infect Control Hosp Epidemiol.* 1992; 13: 606-608
39. Jones VJ. The use of gauze: will it ever change? *Int Wound J*. 2006; 3: 79-86
